# 5,8-Dibromo-15-nitro-2,11-dithia­[3.3]paracyclo­phane

**DOI:** 10.1107/S1600536811048434

**Published:** 2011-11-19

**Authors:** Fang Hu, Zhi-Ming Chen

**Affiliations:** a Key Laboratory of Pesticide and Chemical Biology of the Ministry of Education, College of Chemistry, Central China Normal University, Wuhan 430079, People’s Republic of China

## Abstract

In the title compound [systematic name: 13,15-dibromo-6-nitro-3,10-dithia­tricyclo­[10.2.2.2^5,8^]octa­deca-1(14),5,7,12,15,17-hexa­ene], C_16_H_13_Br_2_NO_2_S_2_, the dihedral angle between the two benzene rings is 0.93 (2)°. The crystal structure is stabilized by weak π–π inter­molecular inter­actions [centroid–centroid distance = 3.286 (5) Å]. One S atom and the H atoms on neighboring C atoms are disordered over two sets of sites (occupancy ratios: S = 0.91:0.09 and H = 0.93:0.07).

## Related literature

For industrial applications of paracyclo­phanes, see: Xu *et al.* (2008 ▶). For the preparation of the title compound, see: Wang *et al.* (2006 ▶).
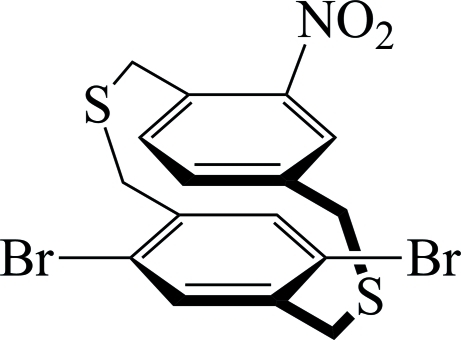


## Experimental

### 

#### Crystal data

C_16_H_13_Br_2_NO_2_S_2_
*M* *_r_* = 475.21
Monoclinic, 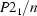

*a* = 6.9200 (3) Å
*b* = 12.6556 (6) Å
*c* = 18.8743 (8) Å
β = 94.939 (2)°
*V* = 1646.81 (13) Å^3^
*Z* = 4
Mo *K*α radiation
μ = 5.18 mm^−1^
*T* = 298 K
0.20 × 0.10 × 0.10 mm

#### Data collection

Bruker SMART CCD area-detector diffractometer
11528 measured reflections
3885 independent reflections
2405 reflections with *I* > 2σ(*I*)
*R*_int_ = 0.046

#### Refinement

*R*[*F* ^2^ > 2σ(*F* ^2^)] = 0.047
*wR*(*F* ^2^) = 0.119
*S* = 0.99
3885 reflections
218 parameters
10 restraints
H-atom parameters constrained
Δρ_max_ = 0.68 e Å^−3^
Δρ_min_ = −0.34 e Å^−3^

### 

Data collection: *SMART* (Bruker, 2001 ▶); cell refinement: *SAINT-Plus* (Bruker, 2001 ▶); data reduction: *SAINT-Plus*; program(s) used to solve structure: *SHELXS97* (Sheldrick, 2008 ▶); program(s) used to refine structure: *SHELXS97* (Sheldrick, 2008 ▶); molecular graphics: *PLATON* (Spek, 2009 ▶); software used to prepare material for publication: *PLATON*.

## Supplementary Material

Crystal structure: contains datablock(s) global, I. DOI: 10.1107/S1600536811048434/jj2107sup1.cif

Structure factors: contains datablock(s) I. DOI: 10.1107/S1600536811048434/jj2107Isup2.hkl

Supplementary material file. DOI: 10.1107/S1600536811048434/jj2107Isup3.cml

Additional supplementary materials:  crystallographic information; 3D view; checkCIF report

## Figures and Tables

**Fig. 1.**
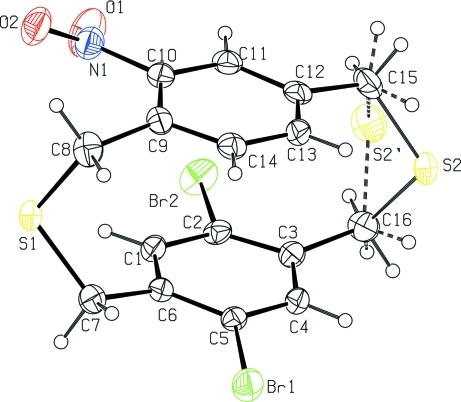
Molecular structure of (I), showing the atom-numbering scheme. Displacement ellipsoids are drawn at the 30% probability level. Dashed lines indicate disorder in the S2 ((0.91:0.09), H15A, H15B (0.93) H15C, H15D (0.07), H16A, H16B (0.93), H16C, H16D (0.07) atoms disordered over two sites.

## supplementary crystallographic information

### Comment

Molecular building blocks associated with *para*-cyclophanes are widely used in chiral catalysis, optoelectronic (NLO) materials, polymer chemistry, materials science, molecular electronics and organic solar cells (Xu *et al.* 2008). Crystallographic studies on these types of complexes are relatively sparse when compared to the volume of synthesis research carried out in these areas. Herein, we report the crystal structure of the title complex, (I).

In the title compound, C_16_ H_13_ Br_2_N O_2_ S_2_, (I), the dihedral angle between the two benzene rings is 0.93 (2)° (Fig. 1). Crystal packing is stabilized by weak π—π intermolecular interactions (centroid-to-centroid distance = 3.286 (5) Å). The S2 atom ((0.91:0.09 for the major and minor components) and H15A, H15B (0.93) H15C, H15D (0.07), H16A, H16B (0.93), H16C, H16D (0.07) atoms are disordered over two sites.

### Experimental

A solution with equimolar amounts of 2,5-dibromo-1,4-bis(mercaptomethyl)benzene (3.26 g,10 mmol) and 1,4-dibromomethyl-2-nitrobenzene(3.10 g,10 mmol) in degassed THF (500 ml) was added dropwise under N_2_ over 12 h to a refluxing solution of potassium carbonate (6.9 g,50 mmol) in EtOH(1.5*L*). After 2 h at the reflux temperature, the mixture was cooled and the solvent removed. The resulting residue was treated with CH_2_Cl_2_ (300 ml) and water (300 ml). The aqueous was extracted with CH_2_Cl_2_ three times. The combined organic layers were dried over Na_2_SO_4_. The solvent was removed, and the resulting solid was chromatographed on silica gel using CH_2_Cl_2_/petroleum ether (1:1, *v*/*v*) as eluent. Colourless single crystals of the title compound suitable for X-ray diffraction were obtained by slow evaporation of a dichloromethane/n-hexane(1:30) solution over a period of 5 days (Wang *et al.*, 2006).

### Refinement

During refinement, all the H atoms were placed in calculated positions and allowed to ride, with CH = 0.93 Å; CH_2_ = 0.97Å and *U*_iso_(H) = 1.2*U*_eq_(C). The S2, C15 & C16 atoms with attached H atoms are disorderd over two sites (occupancies = S: 0.91:0.09; C & H: 0.93: 0.07 for the major and minor components).

### Crystal data

**Table:**

C_16_H_13_Br_2_NO_2_S_2_	*F*(000) = 936
*M**_r_* = 475.21	*D*_x_ = 1.917 Mg m^−^^3^*D*_m_ = 1.917 Mg m^−^^3^*D*_m_ measured by not measured
Monoclinic, *P*2_1_/*n*	Mo *K*α radiation, λ = 0.71073 Å
Hall symbol: -P 2yn	Cell parameters from 1981 reflections
*a* = 6.9200 (3) Å	θ = 2.7–23.5°
*b* = 12.6556 (6) Å	µ = 5.18 mm^−^^1^
*c* = 18.8743 (8) Å	*T* = 298 K
β = 94.939 (2)°	Block, colorless
*V* = 1646.81 (13) Å^3^	0.20 × 0.10 × 0.10 mm
*Z* = 4	

### Data collection

**Table:**

Bruker SMART CCD area-detector diffractometer	2405 reflections with *I* > 2σ(*I*)
Radiation source: fine-focus sealed tube	*R*_int_ = 0.046
graphite	θ_max_ = 28.3°, θ_min_ = 1.9°
Detector resolution: 14.63 pixels mm^-1^	*h* = −6→8
phi and ω scans	*k* = −16→16
11528 measured reflections	*l* = −24→24
3885 independent reflections	

### Refinement

**Table:**

Refinement on *F*^2^	Primary atom site location: structure-invariant direct methods
Least-squares matrix: full	Secondary atom site location: difference Fourier map
*R*[*F*^2^ > 2σ(*F*^2^)] = 0.047	Hydrogen site location: inferred from neighbouring sites
*wR*(*F*^2^) = 0.119	H-atom parameters constrained
*S* = 0.99	*w* = 1/[σ^2^(*F*_o_^2^) + (0.0561*P*)^2^] where *P* = (*F*_o_^2^ + 2*F*_c_^2^)/3
3885 reflections	(Δ/σ)_max_ = 0.001
218 parameters	Δρ_max_ = 0.68 e Å^−^^3^
10 restraints	Δρ_min_ = −0.34 e Å^−^^3^

### Special details

**Table:**

Geometry. All e.s.d.'s (except the e.s.d. in the dihedral angle between two l.s. planes) are estimated using the full covariance matrix. The cell e.s.d.'s are taken into account individually in the estimation of e.s.d.'s in distances, angles and torsion angles; correlations between e.s.d.'s in cell parameters are only used when they are defined by crystal symmetry. An approximate (isotropic) treatment of cell e.s.d.'s is used for estimating e.s.d.'s involving l.s. planes.
Refinement. Refinement of *F*^2^ against ALL reflections. The weighted *R*-factor *wR* and goodness of fit *S* are based on *F*^2^, conventional *R*-factors *R* are based on *F*, with *F* set to zero for negative *F*^2^. The threshold expression of *F*^2^ > σ(*F*^2^) is used only for calculating *R*-factors(gt) *etc*. and is not relevant to the choice of reflections for refinement. *R*-factors based on *F*^2^ are statistically about twice as large as those based on *F*, and *R*- factors based on ALL data will be even larger.

### Fractional atomic coordinates and isotropic or equivalent isotropic displacement parameters (Å^2^)

**Table:**

	*x*	*y*	*z*	*U*_iso_*/*U*_eq_	Occ. (<1)
Br1	0.13008 (8)	0.55071 (3)	0.35467 (3)	0.05578 (18)	
Br2	0.15356 (8)	0.04434 (3)	0.41644 (3)	0.06213 (19)	
S1	0.44323 (16)	0.30062 (8)	0.21060 (5)	0.0411 (3)	
S2	0.34926 (19)	0.28334 (11)	0.59074 (6)	0.0500 (3)	0.91
S2'	0.3416 (13)	0.1428 (9)	0.5658 (8)	0.070 (4)	0.09
O1	0.6200 (6)	0.0274 (3)	0.3297 (2)	0.0847 (12)	
O2	0.7893 (5)	0.1268 (3)	0.26556 (17)	0.0686 (10)	
N1	0.6927 (6)	0.1124 (3)	0.3165 (2)	0.0526 (10)	
C1	0.1908 (6)	0.2278 (3)	0.3351 (2)	0.0373 (10)	
H1	0.2078	0.1787	0.2996	0.045*	
C2	0.1618 (5)	0.1933 (3)	0.4029 (2)	0.0359 (9)	
C3	0.1406 (6)	0.2625 (3)	0.4590 (2)	0.0383 (10)	
C4	0.1303 (6)	0.3690 (3)	0.4410 (2)	0.0382 (10)	
H4	0.1068	0.4182	0.4758	0.046*	
C5	0.1538 (5)	0.4037 (3)	0.3736 (2)	0.0329 (9)	
C6	0.1946 (6)	0.3345 (3)	0.3197 (2)	0.0351 (9)	
C7	0.2498 (6)	0.3724 (3)	0.2476 (2)	0.0461 (11)	
H7A	0.2874	0.4461	0.2519	0.055*	
H7B	0.1355	0.3687	0.2141	0.055*	
C8	0.6582 (6)	0.3413 (4)	0.2651 (2)	0.0490 (11)	
H8A	0.7714	0.3131	0.2447	0.059*	
H8B	0.6671	0.4178	0.2640	0.059*	
C9	0.6617 (6)	0.3057 (3)	0.3415 (2)	0.0388 (10)	
C10	0.6674 (6)	0.2011 (3)	0.3653 (2)	0.0399 (10)	
C11	0.6354 (6)	0.1726 (3)	0.4341 (2)	0.0414 (10)	
H11	0.6364	0.1017	0.4470	0.050*	
C12	0.6017 (5)	0.2495 (4)	0.4840 (2)	0.0405 (10)	
C13	0.6116 (6)	0.3537 (3)	0.4629 (2)	0.0414 (10)	
H13	0.5988	0.4067	0.4963	0.050*	
C14	0.6398 (5)	0.3814 (3)	0.3939 (2)	0.0406 (10)	
H14	0.6443	0.4527	0.3820	0.049*	
C15	0.5534 (6)	0.2184 (4)	0.5567 (2)	0.0565 (13)	
H15A	0.6666	0.2314	0.5895	0.068*	0.93
H15B	0.5293	0.1429	0.5567	0.068*	0.93
H15C	0.5438	0.2820	0.5849	0.068*	0.07
H15D	0.6630	0.1785	0.5782	0.068*	0.07
C16	0.1441 (6)	0.2292 (4)	0.5353 (2)	0.0507 (12)	
H16A	0.1491	0.1527	0.5378	0.061*	0.93
H16B	0.0247	0.2518	0.5541	0.061*	0.93
H16C	0.0229	0.1934	0.5417	0.061*	0.07
H16D	0.1498	0.2914	0.5655	0.061*	0.07

### Atomic displacement parameters (Å^2^)

**Table:**

	*U*^11^	*U*^22^	*U*^33^	*U*^12^	*U*^13^	*U*^23^
Br1	0.0760 (4)	0.0390 (2)	0.0522 (3)	0.0092 (2)	0.0046 (3)	−0.0008 (2)
Br2	0.0733 (4)	0.0420 (3)	0.0746 (4)	0.0017 (2)	0.0269 (3)	0.0120 (2)
S1	0.0484 (7)	0.0486 (6)	0.0270 (6)	0.0011 (5)	0.0074 (5)	−0.0040 (5)
S2	0.0590 (9)	0.0652 (8)	0.0267 (6)	0.0065 (6)	0.0086 (6)	−0.0006 (6)
S2'	0.084 (8)	0.076 (7)	0.052 (7)	0.002 (7)	0.014 (6)	0.020 (6)
O1	0.103 (3)	0.061 (2)	0.095 (3)	−0.026 (2)	0.033 (2)	−0.029 (2)
O2	0.071 (3)	0.082 (3)	0.056 (2)	0.0060 (19)	0.0234 (19)	−0.0144 (18)
N1	0.048 (3)	0.060 (3)	0.051 (3)	−0.001 (2)	0.006 (2)	−0.013 (2)
C1	0.036 (2)	0.040 (2)	0.036 (2)	0.0027 (18)	0.0072 (18)	−0.0042 (18)
C2	0.031 (2)	0.039 (2)	0.040 (2)	0.0043 (17)	0.0125 (18)	0.0083 (19)
C3	0.028 (2)	0.054 (2)	0.034 (2)	0.0013 (19)	0.0103 (18)	0.0036 (19)
C4	0.035 (2)	0.048 (2)	0.032 (2)	0.0038 (19)	0.0054 (18)	−0.0049 (19)
C5	0.028 (2)	0.037 (2)	0.035 (2)	0.0006 (17)	0.0042 (17)	0.0004 (17)
C6	0.030 (2)	0.043 (2)	0.032 (2)	0.0032 (17)	0.0005 (17)	−0.0022 (17)
C7	0.054 (3)	0.051 (3)	0.034 (2)	0.009 (2)	0.007 (2)	0.007 (2)
C8	0.047 (3)	0.057 (3)	0.045 (3)	−0.015 (2)	0.012 (2)	0.000 (2)
C9	0.023 (2)	0.060 (3)	0.033 (2)	−0.0078 (19)	0.0046 (17)	−0.001 (2)
C10	0.031 (2)	0.047 (2)	0.041 (3)	0.0025 (19)	−0.0004 (19)	−0.004 (2)
C11	0.029 (2)	0.052 (3)	0.043 (3)	0.0052 (19)	0.0017 (19)	0.007 (2)
C12	0.020 (2)	0.061 (3)	0.039 (2)	0.0014 (19)	−0.0037 (18)	0.002 (2)
C13	0.033 (3)	0.055 (2)	0.036 (2)	0.004 (2)	0.0006 (19)	−0.010 (2)
C14	0.029 (2)	0.048 (2)	0.044 (3)	−0.0035 (19)	−0.0013 (19)	−0.001 (2)
C15	0.054 (3)	0.082 (3)	0.032 (2)	0.008 (3)	−0.004 (2)	0.011 (2)
C16	0.040 (3)	0.070 (3)	0.044 (3)	0.000 (2)	0.011 (2)	0.013 (2)

### Geometric parameters (Å, °)

**Table:**

Br1—C5	1.899 (4)	C7—H7A	0.9700
Br2—C2	1.904 (4)	C7—H7B	0.9700
S1—C7	1.807 (4)	C8—C9	1.509 (5)
S1—C8	1.809 (4)	C8—H8A	0.9700
S2—C15	1.800 (4)	C8—H8B	0.9700
S2—C16	1.823 (4)	C9—C14	1.396 (6)
S2—H15C	1.3601	C9—C10	1.398 (5)
S2—H16D	1.4245	C10—C11	1.384 (6)
S2'—C15	1.771 (9)	C11—C12	1.386 (6)
S2'—C16	1.806 (9)	C11—H11	0.9300
O1—N1	1.222 (5)	C12—C13	1.381 (6)
O2—N1	1.231 (5)	C12—C15	1.493 (6)
N1—C10	1.472 (5)	C13—C14	1.378 (5)
C1—C6	1.382 (5)	C13—H13	0.9300
C1—C2	1.383 (5)	C14—H14	0.9300
C1—H1	0.9300	C15—H15A	0.9700
C2—C3	1.392 (5)	C15—H15B	0.9700
C3—C4	1.390 (6)	C15—H15C	0.9700
C3—C16	1.499 (5)	C15—H15D	0.9700
C4—C5	1.369 (5)	C16—H16A	0.9700
C4—H4	0.9300	C16—H16B	0.9700
C5—C6	1.389 (5)	C16—H16C	0.9700
C6—C7	1.523 (6)	C16—H16D	0.9700
			
C7—S1—C8	103.8 (2)	C13—C12—C15	122.5 (4)
C15—S2—C16	102.7 (2)	C11—C12—C15	120.2 (4)
C16—S2—H15C	132.5	C14—C13—C12	122.0 (4)
C15—S2—H16D	132.9	C14—C13—H13	119.0
H15C—S2—H16D	155.6	C12—C13—H13	119.0
C15—S2'—C16	104.6 (5)	C13—C14—C9	121.8 (4)
O1—N1—O2	123.4 (4)	C13—C14—H14	119.1
O1—N1—C10	118.0 (4)	C9—C14—H14	119.1
O2—N1—C10	118.6 (4)	C12—C15—S2'	119.0 (6)
C6—C1—C2	120.7 (4)	C12—C15—S2	116.9 (3)
C6—C1—H1	119.6	S2'—C15—S2	62.0 (5)
C2—C1—H1	119.6	C12—C15—H15A	108.1
C1—C2—C3	122.5 (4)	S2'—C15—H15A	131.2
C1—C2—Br2	116.4 (3)	S2—C15—H15A	108.1
C3—C2—Br2	121.0 (3)	C12—C15—H15B	108.1
C4—C3—C2	115.5 (4)	S2'—C15—H15B	47.5
C4—C3—C16	120.3 (4)	S2—C15—H15B	108.1
C2—C3—C16	124.0 (4)	H15A—C15—H15B	107.3
C5—C4—C3	122.1 (4)	C12—C15—H15C	108.5
C5—C4—H4	119.0	S2'—C15—H15C	107.4
C3—C4—H4	119.0	S2—C15—H15C	48.1
C4—C5—C6	121.7 (4)	H15A—C15—H15C	66.4
C4—C5—Br1	118.3 (3)	H15B—C15—H15C	143.0
C6—C5—Br1	120.0 (3)	C12—C15—H15D	107.0
C1—C6—C5	116.9 (4)	S2'—C15—H15D	107.6
C1—C6—C7	120.5 (4)	S2—C15—H15D	134.3
C5—C6—C7	122.6 (4)	H15B—C15—H15D	67.4
C6—C7—S1	116.0 (3)	H15C—C15—H15D	107.0
C6—C7—H7A	108.3	C3—C16—S2'	115.2 (6)
S1—C7—H7A	108.3	C3—C16—S2	113.1 (3)
C6—C7—H7B	108.3	S2'—C16—S2	60.9 (4)
S1—C7—H7B	108.3	C3—C16—H16A	109.0
H7A—C7—H7B	107.4	S2'—C16—H16A	50.0
C9—C8—S1	113.8 (3)	S2—C16—H16A	109.0
C9—C8—H8A	108.8	C3—C16—H16B	109.0
S1—C8—H8A	108.8	S2'—C16—H16B	135.0
C9—C8—H8B	108.8	S2—C16—H16B	109.0
S1—C8—H8B	108.8	H16A—C16—H16B	107.8
H8A—C8—H8B	107.7	C3—C16—H16C	108.0
C14—C9—C10	115.1 (4)	S2'—C16—H16C	108.4
C14—C9—C8	118.6 (4)	S2—C16—H16C	138.0
C10—C9—C8	126.0 (4)	H16A—C16—H16C	63.6
C11—C10—C9	123.1 (4)	H16B—C16—H16C	47.1
C11—C10—N1	115.3 (4)	C3—C16—H16D	109.4
C9—C10—N1	121.5 (4)	S2'—C16—H16D	108.2
C10—C11—C12	120.3 (4)	S2—C16—H16D	50.8
C10—C11—H11	119.9	H16A—C16—H16D	141.5
C12—C11—H11	119.9	H16B—C16—H16D	62.6
C13—C12—C11	117.3 (4)	H16C—C16—H16D	107.4
			
C6—C1—C2—C3	−1.8 (6)	O1—N1—C10—C9	−150.2 (4)
C6—C1—C2—Br2	178.6 (3)	O2—N1—C10—C9	31.4 (6)
C1—C2—C3—C4	6.4 (6)	C9—C10—C11—C12	−2.1 (6)
Br2—C2—C3—C4	−174.0 (3)	N1—C10—C11—C12	−177.4 (3)
C1—C2—C3—C16	−168.8 (4)	C10—C11—C12—C13	−3.2 (6)
Br2—C2—C3—C16	10.8 (6)	C10—C11—C12—C15	175.7 (4)
C2—C3—C4—C5	−4.6 (6)	C11—C12—C13—C14	4.5 (6)
C16—C3—C4—C5	170.8 (4)	C15—C12—C13—C14	−174.3 (4)
C3—C4—C5—C6	−1.8 (6)	C12—C13—C14—C9	−0.6 (6)
C3—C4—C5—Br1	178.4 (3)	C10—C9—C14—C13	−4.5 (6)
C2—C1—C6—C5	−4.7 (6)	C8—C9—C14—C13	169.8 (4)
C2—C1—C6—C7	172.8 (4)	C13—C12—C15—S2'	117.9 (6)
C4—C5—C6—C1	6.5 (6)	C11—C12—C15—S2'	−60.9 (6)
Br1—C5—C6—C1	−173.7 (3)	C13—C12—C15—S2	46.7 (5)
C4—C5—C6—C7	−171.0 (4)	C11—C12—C15—S2	−132.2 (4)
Br1—C5—C6—C7	8.8 (5)	C16—S2'—C15—C12	−65.4 (9)
C1—C6—C7—S1	−38.9 (5)	C16—S2'—C15—S2	41.5 (5)
C5—C6—C7—S1	138.6 (3)	C16—S2—C15—C12	69.6 (4)
C8—S1—C7—C6	−72.3 (4)	C16—S2—C15—S2'	−40.6 (5)
C7—S1—C8—C9	65.9 (4)	C4—C3—C16—S2'	−127.1 (6)
S1—C8—C9—C14	−109.7 (4)	C2—C3—C16—S2'	47.9 (7)
S1—C8—C9—C10	64.0 (5)	C4—C3—C16—S2	−59.7 (5)
C14—C9—C10—C11	5.9 (6)	C2—C3—C16—S2	115.3 (4)
C8—C9—C10—C11	−168.0 (4)	C15—S2'—C16—C3	62.2 (9)
C14—C9—C10—N1	−179.1 (4)	C15—S2'—C16—S2	−41.3 (5)
C8—C9—C10—N1	7.0 (6)	C15—S2—C16—C3	−66.9 (4)
O1—N1—C10—C11	25.2 (6)	C15—S2—C16—S2'	40.2 (5)
O2—N1—C10—C11	−153.2 (4)		
